# First- versus Third-Generation EGFR Tyrosine Kinase Inhibitors in EGFR-Mutated Non-Small Cell Lung Cancer Patients with Brain Metastases

**DOI:** 10.3390/cancers15082382

**Published:** 2023-04-20

**Authors:** Vineeth Tatineni, Patrick J. O’Shea, Ahmad Ozair, Atulya A. Khosla, Shreya Saxena, Yasmeen Rauf, Xuefei Jia, Erin S. Murphy, Samuel T. Chao, John H. Suh, David M. Peereboom, Manmeet S. Ahluwalia

**Affiliations:** 1 Rosa Ella Burkhardt Brain Tumor & Neuro-Oncology Center, Cleveland Clinic, Cleveland, OH 44195, USA; 2 School of Medicine, Case Western Reserve University, Cleveland, OH 44106, USA; 3 Miami Cancer Institute, Baptist Health South Florida, Miami, FL 33176, USA; 4 Division of Neuro-Oncology, University of North Carolina, Chapel Hill, NC 27514, USA; 5 Department of Medical Oncology, Taussig Cancer Institute, Cleveland Clinic, Cleveland, OH 44106, USA; 6 Department of Radiation Oncology, Taussig Cancer Institute, Cleveland Clinic, Cleveland, OH 44106, USA; 7 Herbert Wertheim College of Medicine, Florida International University, Miami, FL 33199, USA

**Keywords:** brain tumor, brain metastasis, lung cancer, lung malignancy, progression-free survival, epidermal growth factor receptor

## Abstract

**Simple Summary:**

Targeted therapies have emerged as newer systemic options for certain cancers. EGFR-directed Tyrosine Kinase Inhibitors (EGFR-TKIs), which have several generations, have been found effective in a type of lung cancer called non-small cell lung cancer (NSCLC) when compared to conventional, platinum-based chemotherapy. More recently, EGFR-TKIs have shown promise in those NSCLC patients where the tumor has developed brain metastases. However, first-generation EGFR-TKIs and novel EGFR-TKIs have also been shown to differ regarding blood-brain-barrier penetration and mutation resistance. In this study, we analyzed the differences between the two generations of EGFR-TKIs in NSCLC patients with brain metastases. Our work did not find differences in overall survival and progression-free survival between the two generations of EGFR-TKIs. However, being a retrospective and single institutional analysis, this study had some limitations, which may have led to an underpowered comparison.

**Abstract:**

**Introduction:** Up to 50% of non-small cell lung cancer (NSCLC) harbor EGFR alterations, the most common etiology behind brain metastases (BMs). First-generation EGFR-directed tyrosine kinase inhibitors (EGFR-TKI) are limited by blood-brain barrier penetration and T790M tumor mutations, wherein third-generation EGFR-TKIs, like Osimertinib, have shown greater activity. However, their efficacy has not been well-studied in later therapy lines in NSCLC patients with BMs (NSCLC-BM). We sought to compare outcomes of NSCLC-BM treated with either first- or third-generation EGFR-TKIs in first-line and 2nd-to-5th-line settings. **Methods:** A retrospective review of NSCLC-BM patients diagnosed during 2010–2019 at Cleveland Clinic, Ohio, US, a quaternary-care center, was performed and reported following ‘strengthening the reporting of observational studies in epidemiology’ (STROBE) guidelines. Data regarding socio-demographic, histopathological, molecular characteristics, and clinical outcomes were collected. Primary outcomes were median overall survival (mOS) and progression-free survival (mPFS). Multivariable Cox proportional hazards modeling and propensity score matching were utilized to adjust for confounders. **Results:** 239 NSCLC-BM patients with EGFR alterations were identified, of which 107 received EGFR-TKIs after diagnosis of BMs. 77.6% (83/107) received it as first-line treatment, and 30.8% (33/107) received it in later (2nd–5th) lines of therapy, with nine patients receiving it in both settings. 64 of 107 patients received first-generation (erlotinib/gefitinib) TKIs, with 53 receiving them in the first line setting and 13 receiving it in the 2nd–5th lines of therapy. 50 patients received Osimertinib as third-generation EGFR-TKI, 30 in first-line, and 20 in the 2nd–5th lines of therapy. Univariable analysis in first-line therapy demonstrated mOS of first- and third-generation EGFR-TKIs as 18.2 and 19.4 months, respectively (*p* = 0.57), while unadjusted mPFS of first- and third-generation EGFR-TKIs was 9.3 and 13.8 months, respectively (*p* = 0.14). In 2nd–5th line therapy, for first- and third-generation EGFR-TKIs, mOS was 17.3 and 11.9 months, (*p* = 0.19), while mPFS was 10.4 and 6.08 months, respectively (*p* = 0.41). After adjusting for age, performance status, presence of extracranial metastases, whole-brain radiotherapy, and presence of leptomeningeal metastases, hazard ratio (HR) for OS was 1.25 (95% CI 0.63–2.49, *p* = 0.52) for first-line therapy. Adjusted HR for mOS in 2nd-to-5th line therapy was 1.60 (95% CI 0.55–4.69, *p* = 0.39). **Conclusions:** No difference in survival was detected between first- and third-generation EGFR-TKIs in either first or 2nd-to-5th lines of therapy. Larger prospective studies are warranted reporting intracranial lesion size, EGFR alteration and expression levels in primary tumor and brain metastases, and response rates.

## 1. Introduction

Lung cancer is the second most common type of cancer worldwide and the leading cause of cancer-related mortality in both male and female adults [1]. Non-small cell lung cancer (NSCLC) accounts for 80% of all lung cancers and is the most common cause of brain metastases [2]. With nearly 10–30% of patients with NSCLC developing brain metastasis, contributing to poorer prognosis and more symptoms, research in the field of brain metastasis has dramatically increased over the last decade [3].

In more recent years, the management of NSCLC has shifted from platinum-based chemotherapy to targeted molecular therapies. While multiple immunohistochemical markers have been studied, only a handful have been shown to be reliable targets and prognostic markers [3]. Epidermal growth factor receptor (EGFR) is a transmembrane receptor tyrosine kinase that is mutated in 40% to 60% of NSCLC with brain metastasis (NSCLC-BM) [4]. The signaling pathway, prompted by several growth factors, leads to autophosphorylation, causes tumor proliferation, and boosts cell survival [5]. The risk of developing brain metastases is higher in EGFR-altered patients, though providentially, the EGFR signaling pathway is being increasingly targeted [6]. There exist multiple known EGFR-related mutations, including deletion of exon 18, deletion of exon 19, exon 21-point mutation, and exon 20 insertion mutation [5]. Different mutations cause different structural alterations in the EGFR protein, which leads to differential sensitivities from targeted therapies [7].

While there exist multiple treatment options for treating NSCLC-BM, including whole-brain radiotherapy (WBRT), stereotactic radiosurgery (SRS), and, more rarely, surgical resection, the standard of care has shifted to the use of EGFR-directed tyrosine kinase inhibitors (EGFR-TKIs) [8,9,10]. EGFR-TKIs are reversible TKI inhibitors that target the adenosine triphosphate (ATP) cleft within the receptor [11]. First-generation EGFR-TKIs, such as erlotinib and gefitinib, were introduced in the early 2000s and have proven more effective than standard chemotherapy [12,13]. However, the efficacy of first-generation EGFR-TKIs for treating NSCLC-BM is limited by blood-brain barrier (BBB) penetration and exon 20 (T790M) tumor mutations [14,15]. Previous reports have shown the cerebrospinal fluid (CSF) concentration levels of first-generation EGFR-TKIs were low when given standard doses [5]. Though higher concentration levels could be achieved with higher doses, their peak was short-lived [5,15]. More frequent dosing, from weekly to daily, was also tested but was associated with more toxicity [16]. Even in patients with good responses to first-generation EGFR-TKIs, efficacy may be lost due to acquired resistance from T790M mutations [17]. Third-generation EGFR-TKIs, such as Osimertinib, introduced in the mid-2010s, have shown better BBB penetration and efficacy against T790M mutations [18].

Multiple studies, including the FLAURA and OCEAN trials, have demonstrated the efficacy of Osimertinib in NSCLC-BM. Data from the initial FLAUR publication and its follow-up demonstrated improved progression-free survival (PFS) and overall survival (OS) with Osimertinib compared to first-generation EGFR-TKIs. These findings have led to Osimertinib being increasingly used as first-line treatment in patients with EGFR-mutant NSCLC and NSCLC-BM [19,20]. These studies still leave a gap in comparing the efficacy of EGFR-TKI when given as first-line versus later-line therapies. Given the limited data and publications, we sought to compare the OS and PFS in NSCLC-BM patients treated with first versus third-generation EGFR-TKIs, in both first and later-line therapies.

## 2. Methods

### 2.1. Patient Selection and Data Collection

A retrospective cohort study involving EGFR-altered NSCLC-BM patients treated at Cleveland Clinic (Cleveland, OH, USA), a quaternary-care institution, was conducted and reported following ‘strengthening the reporting of observational studies in epidemiology’ (STROBE) guidelines. The work was approved by the Cleveland Clinic, Ohio Institutional Review Board (reference number 09-911) before commencement. Inclusion criteria for our study included all patients ≥18 years of age with EGFR-altered NSCLC-BM treated with erlotinib, gefitinib, or Osimertinib at any point after the diagnosis of brain metastases from 2010 to 2019 at our institution.

Patient demographics, initial diagnostic and genomic testing information, and treatment details were collected from the institution’s electronic medical record. Among the information collected was Karnofsky Performance Score (KPS), age, race, and sex. Collected treatment details include the date of initiation, date of progress, line of therapy, and generation of EGFR inhibitor used. Data was recorded in REDCap, a secure database. Patients included in our study were followed in the outpatient setting every three months. The start of a new line of therapy, the use of SRS during EGFR inhibitor treatment, or death were also used to define disease progression. In patients with questionable pseudo-progression, the case was assessed at the hospital’s interdisciplinary tumor board.

### 2.2. EGFR-TKI Data

Only patients treated with erlotinib, gefitinib, and Osimertinib were primarily investigated in this study. Treatment with first- versus third-generation EGFR-TKIs was primarily due to temporal effects. Third-generation TKIs are increasingly utilized as relevant literature, and recommendations gradually accumulated regarding their utility. We did not exclude patients who received erlotinib and gefitinib prior to the diagnosis of brain metastases if they received Osimertinib after the diagnosis of brain metastases, as we only evaluated response rates after the diagnosis of brain metastases. First-line therapy was defined as EGFR-TKI treatment given as the first systemic therapy after the diagnosis of brain metastases. Later (2nd to 5th) lines of therapy were defined as the initial EGFR-TKI given after the diagnosis of brain metastases but not as the first systemic therapy. Any patients experiencing breaks during the treatment due to symptoms were not excluded as long as there was no progression. We included patients who received EGFR-TKI, then had progression, and later also received EGFR-TKI. We also included patients who were taking EGFR-TKI, then had intracranial progression for which local control was attempted while EGFR-TKI was continued.

### 2.3. Statistical Methods

Categorical clinical and pathologic variables were summarized as frequency counts and percentages. Continuous variables were summarized as medians and ranges. Kruskal-Wallis Tests and Fisher’s exact test was used to compare the quantitative and factor variables among treatment groups. OS was measured from the start date of the first treatment received to the date of the last follow-up or date of death and was summarized using the Kaplan-Meier method. PFS was measured from the start date of the treatment to the start date of a new line of therapy, the start date of the following SRS, or the date of the last follow-up or date of death. 1-year and 2-year survival rates and estimated median survivals for each treatment cohort were reported. Log-rank tests were used for univariable comparisons between treatments. The Cox proportional hazard model with a two-sided Wald test was used to evaluate the impact of the treatment on OS and PFS. The survival model was adjusted by clinical variables selected by the random forest method. The primary model was adjusted by the variables which were mostly identified as prognostic factors in patients with NSCLC-BM in previous studies [20]. These variables were age at diagnosis of brain metastases, gender, number of brain metastases, the existence of extracranial metastases, the existence of leptomeningeal metastases, KPS, and the duration from the date of diagnosis of brain metastases to the date of treatment. The first-generation EGFR-TKI cohort was used as the reference group for comparing OS and PFS due to being the older medication group with a long use history. Propensity score matching was also performed. Statistical significance was defined as a *p*-value of <0.05. All statistical analysis was performed using R Statistical Software version 4.1.0 (R Foundation for Statistical Computing, Vienna, Austria).

## 3. Results

### 3.1. Patient Characteristics

**Figure 1:**
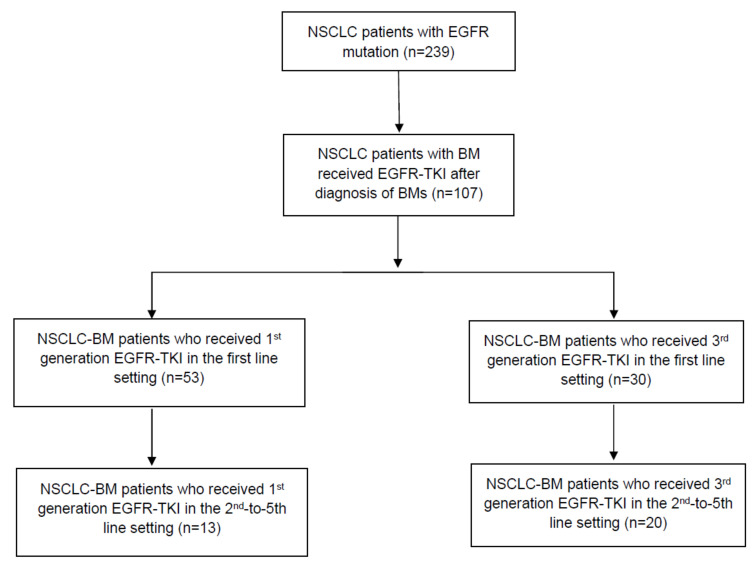
Flow diagram of the current study.

Between 2010 and 2019, we found 239 eligible patients who had NSCLC-BM with an EGFR alteration in the primary tumor. Overall, the median PFS (mPFS) was 6.3 months. The 1-year OS rate for EGFR-positive patients was 68% (95% confidence interval (CI) = 59%, 75%). The 2-year OS rate was 31% (95% CI = 23%, 40%). The 1-year PFS rate for the same overall encompassing group was 34% (95% CI = 27%, 42%), with a 2-year PFS rate of 14% (95% CI = 9%, 21%). The patient population was split into cohorts based on treatment with first-generation EGFR-TKIs and treatment with third-generation EGFR-TKIs (Figure 1, Table 1). 107 EGFR-mutant patients received EGFR-TKIs after diagnosis of BMs. 77.6% (83/107) received it as first-line treatment, and 30.8% (33/107) received it in later (2nd–5th) lines of therapy, with nine patients receiving it in both settings.

A total of 64 of 107 patients received first-generation (erlotinib/gefitinib) TKIs, with 53 receiving them in the first line setting and 13 receiving it in the 2nd–5th line of therapy (Table 2). 50 patients received Osimertinib as third-generation EGFR-TKI, 30 as first-line, and 20 as the 2nd–5th line of therapy. (Table 3). Later-line therapy was defined as systemic therapy given as the 2nd–5th line of therapy. The characteristics of the cohort are separately documented in Table 2 and Table 3.

### 3.2. Overall Survival

**Figure 2:**
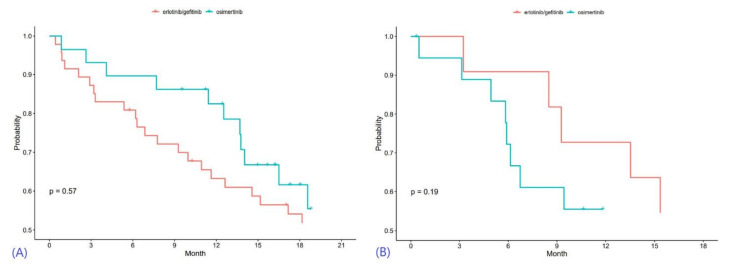
Kaplan-Meier curves for overall survival, with results of first-line therapy in (**A**) and outcomes of later-line therapy in (**B**).

When erlotinib or gefitinib was given as first-line therapy, the unadjusted median OS (mOS) was 18.2 months, while patients given Osimertinib in the first-line setting had an mOS of 19.4 months (Table 4). Kaplan-Meier curves for overall survival are provided in Figure 2. For the erlotinib/gefitinib cohort, the 1-year OS rate was 63% (95% CI 48%, 75%), and the 2-year OS rate was 32% (95% CI = 19%, 46%). The 1-year OS rate for the Osimertinib cohort was 82% (95% CI = 63%, 92%), and the 2-year OS rate for NSCLC-BM patients treated with Osimertinib as first-line therapy was 36% (95% CI = 13%, 61%).

Univariable Cox Proportional Hazards modeling of OS, using both categorical and continuous variables, is demonstrated in Table 5. After adjusting for age, KPS score, extracranial metastases, receipt of WBRT, and leptomeningeal metastases in multivariable analysis, there was no statistically significant OS difference found between the two first-line therapy cohorts (HR 1.25, 95% CI 0.63, 2.49, *p* = 0.52). For the 2nd-to-5th line of therapy, unadjusted comparison demonstrated no significant difference in mOS (*p* = 0.19). Multivariable analysis once again showed no statistical significance in mOS between the two cohorts (HR 1.60. 95% CI 0.55–4.69, *p* = 0.39) (Table 6 and Table 7).

### 3.3. Progression-Free Survival

**Figure 3:**
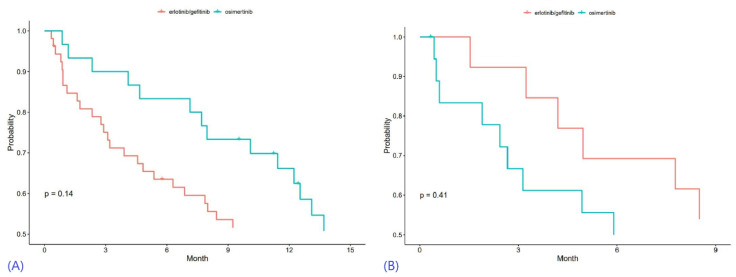
Kaplan-Meier curves for progression-free survival, with results of first-line therapy in (**A**) and outcomes of later-line therapy in (**B**).

For first-line therapy in NSCLC-BM patients, the unadjusted median PFS (mPFS) of first-generation and third-generation EGFR-TKIs was 9.27 months and 13.77 months, respectively, with no significant difference (Table 8). The 1-year PFS rate for first-generation EGFR-TKIs was 41% (95% CI 28%, 55%), while the 2-year PFS rate was 16% (95% CI 7%, 27%). NSCLC-BM patients treated with third-generation EGFR-TKIs as first-line therapy showed a 1-year PFS rate of 66% (95% CI = 46%, 80%) and a 2-year PFS rate of 34% (95% CI = 15%, 55%) (Figure 3).

Univariable Cox Proportional Hazards modeling of OS, using both categorical and continuous variables, is demonstrated in Table 9. There was also no statistically significant difference in PFS between the two cohorts when given as 1st line systemic therapy, with the adjustment of age, sex, extracranial mets, and WBRT treatment (HR 1.0, 95% CI 0.54–1.83, *p* = 0.99). When given as the 2nd to 5th line of systemic therapy in NSCLC-BM patients, the mPFS demonstrated no statistically significant difference (HR 1.12, 95% CI 0.43–2.93; *p* = 0.82) (Table 10 and Table 11).

### 3.4. Outcomes after Propensity Score Matching

Propensity score matching was also performed to reduce baseline confounding, whose results are given in Table 12.

## 4. Discussion

In recent years, novel EGFR inhibitors, specifically Osimertinib, have taken precedence as the first-line treatment for EGFR-altered NSCLC over first-generation EGFR-TKIs [19,20]. In this work, we attempted to evaluate the efficacy of these drugs in NSCLC-BM patients at a single institution. Recent animal studies have shown better BBB penetration with Osimertinib than gefitinib, rociletinib, or afatinib, suggesting Osimertinib may have better survival outcomes in NSCLC-BM patients [18]. However, our study failed to show any statistically significant difference in PFS or OS between novel EGFR-TKI and erlotinib/gefitinib when treating EGFR-altered NSCLC-BM patients, either as first-line treatment or as a later line of treatment.

The FLAURA trial showed a clear survival benefit in NSCLC patients treated with third-generation EGFR-TKIs compared to first-generation EGFR-TKIs [20]. The FLAURA trial included patients with locally advanced or metastatic NSCLC, required to have proof of *EGFR* exon 19 deletions or p.Leu858Arg *EGFR* mutation. This Phase III trial randomized 556 patients in a 1:1 ratio to either Osimertinib or the standard of care (physician’s choice of erlotinib or gefitinib). Osimertinib was found to improve median PFS from 10.2 months with the erlotinib/gefitinib to 18.9 months with Osimertinib (HR 0.46; 95% CI 0.37 to 0.57; *p* < 0.001). More specifically, when a subgroup of 116 patients with CNS metastases was evaluated, median PFS in NSCLC-BM patients treated with Osimertinib treatment (53 patients, PFS 15.2 months) was also found to be significantly higher than those provided the standard of care (63 patients, PFS 9.6 months) (HR 0.47; 95% CI 0.30–0.74; *p* < 0.001). However, the FLAURA trial sub-analysis included patients who were treated previously with intracranial radiation [21,22]. The OCEAN study was a prospective study that evaluated Osimertinib in radiation-naive NSCLC-BM, again showing good efficacy with an overall response rate (ORR) of 40.5% and a median brain metastasis-related PFS of 25.2 months [23]. However, all the participants in the OCEAN trial were previously treated with older EGFR-TKIs [23]. The phase I BLOOM study further demonstrated Osimertinib’s favorable CSF efficacy by analyzing radiological and symptomatic responses in NSCLC with leptomeningeal disease [24,25].

Only a few studies have evaluated the intracranial efficacy of EGFR-TKIs, generally reporting the benefit of 3rd generation EGFR-TKI use. Huang et al. compared the efficacy of Osimertinib and afatinib in treating EGFR-altered NSCLC and NSCLC-BM in the Taiwanese population. Interestingly, they reported a significant increase in PFS using Osimertinib (22.1 months vs. 12.9 months, *p* = 0.045) in patients with brain metastasis. However, there was no difference in median PFS in patients without brain metastasis (HR 1.02, 95% CI 0.56–1.85). When analyzed without subgroups, no statistically significant difference in median progression-free survival was found [26]. In another Asian cohort with NSCLC, Gen et al. studied 388 patients treated with EGFR-TKIs as 1st line therapy at five institutions. In a subgroup analysis of 118 patients with metastatic NSCLC disease in the brain, this study reported a longer PFS with Osimertinib compared to 1st gen TKIs erlotinib/gefitinib and 2nd gen TKI afatinib (16.3 vs. 7.9 vs. 8.3 months respectively). An improvement in OS was also noted to be trending towards significance with the use of Osimertinib compared to erlotinib/gefitinib (not reached vs. 20.9 months, *p* = 0.0725) [27].

Zhao et al. evaluated a Chinese cohort of 367 patients with NSCLC-BM subjected to either first-generation EGFR TKIs or Osimertinib as the first line of treatment. This study demonstrated a superior OS and intracranial ORR with the use of Osimertinib, despite the patients receiving it having a greater number and size of BMs than 1st gen TKIs (37.7 vs. 22.2 months, 68% vs. 50%, respectively) [28]. Meanwhile, Zhou et al. findings from a different approach. They chose a cohort of 813 diagnosed with EGFR-altered NSCLC without baseline CNS metastases who were treated with a 1st gen TKI or Osimertinib. 38 patients in the cohort developed CNS metastasis during treatment. They observed a decrease in risk of subsequent development of CNS metastases in patients treated with Osimertinib vs. 1st gen TKIs gefitinib or erlotinib. However, this result was not statistically significant (*p* = 0.059) [29]. In another study, Reungwetwattana et al. analyzed 200 brain metastases patients as a subset of the FLAURA trial. They found that the median CNS progression-free survival in patients with measurable or non-measurable CNS lesions was not reached with Osimertinib (95% CI, 16.5–NA) and 13.9 months (95% CI, 8.3–NA) with standard EGFR-TKIs (HR 0.48; 95% CI, 0.26–0.86; *p* = 0.014). These results were named nominally statistically significant, and further analysis showed that objective response rates were also improved in the patients receiving Osimertinib [30]. There are not many studies on this issue, and the existing studies have smaller sample sizes than would be ideal to fully elucidate the effect of 3rd generation EGFR TKIs in NSCLC-BM patients, as is our work. An underpowered comparison may partially explain the variability in outcomes, including progression-free survival.

The discrepancy between this work and prior literature may also be due to various reasons. First, our study had a small sample size of the first-line Osimertinib cohort; this led to a much higher median age, a known prognostic variable for brain metastases. However, interestingly the first-line Osimertinib treatment group also had fewer patients with extracranial metastasis and leptomeningeal spread. Secondly, there may have been confounding systemic therapies for EGFR-TKIs analyzed as 2nd to 5th line. Since our study was a retrospective cohort, considerable selection bias was likely present. Multivariable analysis, like the one performed in this work, can only adjust for the known confounders, typically just some of them. Finally, the complexity of defining PFS may have led to a lack of statistical difference between the two cohorts. PFS was defined as SRS after treatment, the start date of the next line of treatment, the date of death, or the date of the last follow-up. No MRI brain metastases measurements were collected in our study, which would have provided the most accurate way to assess tumor progression. Nevertheless, our study provides another important data point in assessing targeted therapies in brain metastases from lung cancer.

Though some studies have shown promise for Osimertinib’s BBB penetration, mutation resistance, and overall efficacy in NSCLC-BM, further studies need to be conducted to show intracranial efficacy by examining MRI measurements [18,19,20]. Large prospective studies are warranted that examine, along with the variables mentioned above, the determination of the genetic alteration(s) (e.g., EGFR) and level of expression in both primary tumors and brain metastasis. EGFR-altered NSCLC-BM treatments continue to evolve, as there are currently ongoing studies with Osimertinib and combination therapy, including SRS or immune checkpoint inhibitors [10,31]. With advances in precision medicine, strategic management approaches in the use of EGFR, especially for lung cancer-related metastasis in neuro-oncology, will continue to change.

## 5. Conclusions

This study found no survival benefit between the novel EGFR-TKIs and first-generation EGFR-TKIs when given either as first-line therapy or an alternative line of therapy in patients with EGFR-altered NSCLC with brain metastases. Larger studies, with rigorous, prospective data collection, are warranted, with reporting for intracranial lesion size, determination of the type of EGFR alteration, and level of EGFR expression in both primary tumors and brain metastases, along with intracranial and extracranial response rates.

## Figures and Tables

**Table 1:** Number of patients with EGFR-mutant NSCLC patients with brain metastases who received EGFR-TKI after BM diagnosis.

Group	*n*	Follow-Up Time
Total EGFR-mutant NSCLC patients treated with EGFR-TKI after BM diagnosis	107	17.1 months
Treated with first-generation EGFR-TKI (erlotinib/gefitinib), *n*	64	18.03 months
Treated with first-generation EGFR-TKI (Osimertinib), *n*	50	17.95 months

**Table 2:** Characteristics of patients with NSCLC brain metastases who received EGFR-directed Tyrosine Kinase Inhibitors (EGFR-TKIs) in the first-line setting.

Variable	Erlotinib/Gefitinib	Osimertinib
Cohort population (*n*)	53	30
Age in years, median (range)	63.1 (29.9, 90.7)	72.77 (31.92, 84.83)
Female, *n* (%)	32 (60.4)	23 (76.7)
KPS ≥70, *n* (%)	48 (96.0)	29 (96.7)
Multiple brain metastases, *n* (%)	42 (84.0)	21 (72.4)
Single brain metastases, *n* (%)	8 (16.0)	8 (27.6)
Extracranial metastases, *n* (%)	42 (80.8)	13 (44.8)
Leptomeningeal spread, *n* (%)	5 (9.4)	1 (3.3)
Received WBRT, *n* (%)	33 (62.3)	7 (23.3)
Received Surgery, *n* (%)	6 (11.3)	1 (3.3)
Received SRS, *n* (%)	29 (54.7)	20 (66.7)
Median Number of SRS (Range)	0 (0–10)	0 (0–8)

KPS, Karnofsky Performance Status; WBRT, Whole Brain Radiotherapy; SRS, Stereotactic Radiosurgery.

**Table 3:** Characteristics of patients with NSCLC brain metastases who received line EGFR-directed Tyrosine Kinase Inhibitors (EGFR-TKIs) in later (2nd to 5th) lines of therapy.

Variable	Erlotinib/Gefitinib	Osimertinib
Cohort population (*n*)	13	20
Age in years, median (range)	59.7 (46.8, 72.8)	62.7 (28.4, 83.5)
Female, *n* (%)	8 (61.5)	11 (55.0)
KPS ≥ 70, *n* (%)	11 (84.6)	19 (100.0)
Multiple brain metastases, *n* (%)	8 (72.7)	15 (78.9)
Single brain metastases, *n* (%)	3 (27.3)	4 (21.1)
Extracranial metastases, *n* (%)	9 (69.2)	18 (90.0)
Leptomeningeal spread, *n* (%)	1 (7.7)	1 (5.0)
Received surgery, *n* (%)	3 (23.1)	1 (5.0)
Received WBRT, *n* (%)	6 (46.2)	11 (55.0)
Received SRS, *n* (%)	9 (69.2)	14 (70.0)
Median Number of SRS (Range)	1 (0–3)	1 (0–5)

KPS, Karnofsky Performance Status; WBRT, Whole Brain Radiotherapy; SRS, Stereotactic Radiosurgery.

**Table 4:** Overall survival (OS) of patients with NSCLC brain metastases treated with first-generation and third-generation EGFR-directed Tyrosine Kinase Inhibitors (EGFR-TKIs) in the first line and 2nd-to-5th line treatment settings. NA: Not Available.

Therapy	EGFR-TKI	Median OS (Months)	1-Year OS (95% CI)	2-Year OS (95% CI)
1st line	erlotinib/gefitinib	18.2	63% (48%, 75%)	32% (19%, 46%)
	Osimertinib	19.4	82% (63%, 92%)	36% (13%, 61%)
2nd–5th line	erlotinib/gefitinib	17.3	NA	NA
	Osimertinib	11.9	NA	NA

**Table 5:** Univariable Cox Proportional Hazards modeling of overall survival.

Variable	Level	1st Line		2nd-to-5th Line	
		HR (95% CI)	*p*-Value	HR (95% CI)	*p*-Value
Number of Brain Metastases	Multiple	Reference		Reference	
	Single	0.67 (0.42, 1.07)	0.09	0.24 (0.05, 1.05)	0.057
Extracranial Metastases Present at Time of Diagnosis	No	Reference		Reference	
	Yes	2.29 (1.44, 3.64)	<0.001	4.23 (0.92, 19.48)	0.064
Leptomeningeal metastases	No	Reference		Reference	
	Yes	1.83 (0.95, 3.52)	0.071	43.10 (3.81, 487.17)	0.002
Whole Brain Radiation Received	No	Reference		Reference	
	Yes	1.83 (1.24, 2.69)	0.002	2.13 (0.89, 5.10)	0.091
Surgery Received	No	Reference		Reference	
	Yes	0.96 (0.55, 1.69)	0.88	0.78 (0.26, 2.40)	0.67
Karnofsky Performance Status	≥70	Reference		Reference	
	70	1.92 (0.92, 4.03)	0.083	0.97 (0.13, 7.39)	0.98
SRS frequency	≥1	Reference		Reference	
	0	1.37 (0.92, 2.04)	0.12	1.24 (0.47, 3.29)	0.66
Sex	Female	Reference		Reference	
	Male	0.98 (0.67, 1.45)	0.93	0.58 (0.23, 1.47)	0.25
Generation of EGFR-TKI received	1st	Reference		Reference	
	3rd	0.84 (0.45, 1.55)	0.57	1.83 (0.74, 4.56)	0.19
Number of Brain metastases	-	1.06 (0.99, 1.12)	0.08	1.07 (0.88, 1.31)	0.51
SRS Total Number	-	0.78 (0.67, 0.92)	0.003	1.05 (0.73, 1.50)	0.79
Age	-	1.00 (0.98, 1.02)	0.99	0.98 (0.95, 1.02)	0.31

**Table 6:** Multivariable Cox-Proportional Hazards Modelling of overall survival (OS) for EGFR-altered NSCLC patients with brain metastases in first-line EGFR-TKI.

Variable	Level	HR (95% CI)	*p*-Value
Karnofsky performance status	≥70	Reference	
	<70	2.03 (0.57, 7.20)	0.28
Extracranial metastases at diagnosis	Absent	Reference	
	Present	3.10 (1.42, 6.76)	0.004
Whole-brain radiotherapy	No	Reference	
	Yes	1.58 (0.83, 3.01)	0.17
Age	-	1.01 (0.99, 1.03)	0.46
Leptomeningeal metastases	Absent	Reference	
	Present	0.71 (0.26, 1.92)	0.50
Generation of EGFR TKI	1st	Reference	
	3rd	1.25 (0.63, 2.49)	0.52

**Table 7:** Multivariable Cox-Proportional Hazards Modeling of overall survival (OS) for EGFR-altered NSCLC patients with brain metastases in 2nd-to-5th-line EGFR-TKI.

Variable	Level	HR (95% CI)	*p*-Value
Leptomeningeal metastases	Absent	Reference	
	Present	26.30 (1.91, 362.80)	0.015
Extracranial metastases at diagnosis	Absent	Reference	
	Present	4.06 (0.62, 26.71)	0.14
Generation of EGFR TKI	erlotinib/gefitinib	Reference	
	Osimertinib	1.60 (0.55, 4.69)	0.39
Sex	Female	Reference	
	Male	0.32 (0.10, 0.97)	0.045
Whole-brain radiotherapy	No	Reference	
	Yes	1.81 (0.62, 5.26)	0.28
Age	-	0.97 (0.93, 1.02)	0.23

**Table 8:** Unadjusted Progression-free survival (PFS) of patients with NSCLC brain metastases treated with first-generation and third-generation EGFR Tyrosine Kinase Inhibitors (EGFR-TKIs). NA: Not Available.

Therapy	EGFR-TKI	Median PFS (Months)	1-Year PFS (95% CI)	2-Year PFS (95% CI)
1st line	erlotinib/gefitinib	9.27	41% (28%, 55%)	16% (7%, 27%)
	Osimertinib	13.77	66% (46%, 80%)	34% (15%, 55%)
2nd–5th line	erlotinib/gefitinib	10.43	NA	NA
	Osimertinib	6.08	NA	NA

**Table 9:** Univariable Cox Proportional Hazards modeling of progression-free survival.

Variable	Level	1st Line		2nd-to-5th Line	
		HR (95% CI)	*p*-Value	HR (95% CI)	*p*-Value
Number of Brain Metastases	Multiple	Reference		Reference	
	Single	1.05 (0.70, 1.55)	0.82	0.20 (0.06, 0.70)	0.012
Extracranial Metastases Present at the time of diagnosis	No	Reference		Reference	
	Yes	1.68 (1.13, 2.49)	0.01	3.19 (0.94, 10.84)	0.064
Leptomeningeal metastases	No	Reference		Reference	
	Yes	0.82 (0.43, 1.53)	0.53	6.19 (1.27, 30.05)	0.024
Whole Brain Radiation Received	No	Reference		Reference	
	Yes	1.38 (0.97, 1.94)	0.07	2.94 (1.31, 6.64)	0.009
Received Surgery	No	Reference		Reference	
	Yes	1.20 (0.70, 2.06)	0.51	0.82 (0.28, 2.42)	0.72
Karnofsky Performance Status	≥70	Reference		Reference	
	70	1.42 (0.74, 2.72)	0.29	0.79 (0.18, 3.39)	0.75
SRS frequency	≥1	Reference		Reference	
	0	0.86 (0.60, 1.24)	0.41	0.73 (0.30, 1.73)	0.47
Sex	Female	Reference		Reference	
	Male	1.55 (1.10, 2.18)	0.013	1.12 (0.51, 2.45)	0.79
Generation of EGFR-TKI received	1st	Reference		Reference	
	3rd	0.66 (0.38, 1.15)	0.14	1.39 (0.63, 3.04)	0.41
Number of Brain metastases	-	1.05 (0.98, 1.12)	0.14	1.03 (0.89, 1.18)	0.72
SRS Total Number	-	0.95 (0.85, 1.07)	0.37	1.19 (0.88, 1.62)	0.25
Age	-	0.99 (0.98, 1.01)	0.29	0.98 (0.95, 1.01)	0.14

**Table 10:** Multivariable Cox-Proportional Hazards Modelling of progression-free survival (PFS) for EGFR-altered NSCLC patients with brain metastases in first-line EGFR-TKI.

Variable	Level	HR (95% CI)	*p*-Value
Sex	Female	Reference	0.016
	Male	1.9 (1.13, 3.23)	
Extracranial metastases at diagnosis	Absent	Reference	0.092
	Present	1.7 (0.91, 3.34)	
Whole-brain radiotherapy received	No	Reference	0.013
	Yes	2.1 (1.17, 3.93)	
Age	-	1.7 (0.91, 3.34)	0.95
Generation of EGFR TKI received	1st	Reference	>0.99
	3rd	1.0 (0.54, 1.83)	

**Table 11:** Multivariable Cox-Proportional Hazards Modelling of progression-free survival for EGFR-altered NSCLC patients with brain metastases in 2nd-to-5th-line EGFR-TKI.

Variable	Level	HR (95% CI)	*p*-Value
Leptomeningeal metastases	Absent	Reference	0.03
	Present	22.14 (1.35, 364.28)	
Extracranial metastases at diagnosis	Absent	Reference	0.35
	Present	1.96 (0.48, 7.98)	
Generation of EGFR TKI Received	1st	Reference	0.82
	3rd	1.12 (0.43, 2.93)	
Brain Metastases	Multiple	Reference	0.046
	Single	0.25 (0.07, 0.98)	
Age	-	1.12 (0.43, 2.93)	0.40

**Table 12:** Propensity score matching between first- and third-generation EGFR-TKI cohorts.

	EGFR-TKI	No. of Obs.	No. of Events	Median Duration (Month)	1-Year Rate (95% CI)	2-Year Rate (95% CI)	HR (95% CI)	*p*
OS	erlotinib/gefitinib	28	23	18.2	64% (44%, 79%)	28% (13%, 46%)	Reference	0.55
	Osimertinib	28	14	23.9	82% (62%, 92%)	38% (13%, 63%)	0.81 (0.40–1.63)	
PFS	erlotinib/gefitinib	28	26	9.37	43% (24%, 60%)	18% (6%, 35%)	Reference	0.26
	Osimertinib	28	17	13.77	64% (43%, 79%)	32% (12%, 55%)	0.69 (0.36–1.31)	

## Data Availability

The study lead authors, and senior authors have access to the primary dataset. Data may be made available to interested investigators upon reasonable request to the corresponding author (manmeeta@baptisthealth.net) after approval by all required regulatory authorities.

## References

[1] Bray F., Ferlay J., Soerjomataram I., Siegel R.L., Torre L.A., Jemal A. (2018). Global cancer statistics 2018: GLOBOCAN estimates of incidence and mortality worldwide for 36 cancers in 185 countries. CA Cancer J. Clin..

[2] Gelatti A.C.Z., Drilon A., Santini F.C. (2019). Optimizing the sequencing of tyrosine kinase inhibitors (TKIs) in epidermal growth factor receptor (EGFR) mutation-positive non-small cell lung cancer (NSCLC). Lung Cancer.

[3] Saad A.G., Yeap B.Y., Thunnissen F.B., Pinkus G.S., Pinkus J.L., Loda M., Sugarbaker D.J., Johnson B.E., Chirieac L.R. (2008). Immunohistochemical markers associated with brain metastases in patients with non-small cell lung carcinoma. Cancer.

[4] Rangachari D., Yamaguchi N., VanderLaan P.A., Folch E., Mahadevan A., Floyd S.R., Uhlmann E.J., Wong E.T., Dahlberg S.E., Huberman M.S. (2015). Brain metastases in patients with EGFR-altered or ALK-rearranged non-small-cell lung cancers. Lung Cancer.

[5] Rybarczyk-Kasiuchnicz A., Ramlau R., Stencel K. (2021). Treatment of Brain Metastases of Non-Small Cell Lung Carcinoma. Int. J. Mol. Sci..

[6] Aiko N., Shimokawa T., Miyazaki K., Misumi Y., Agemi Y., Ishii M., Nakamura Y., Yamanaka T., Okamoto H. (2018). Comparison of the efficacies of first-generation epidermal growth factor receptor tyrosine kinase inhibitors for brain metastasis in patients with advanced non-small-cell lung cancer harboring EGFR mutations. BMC Cancer.

[7] Passaro A., Mok T., Peters S., Popat S., Ahn M.J., de Marinis F. (2021). Recent Advances on the Role of EGFR Tyrosine Kinase Inhibitors in the Management of NSCLC With Uncommon, Non Exon 20 Insertions, EGFR Mutations. J. Thorac. Oncol..

[8] Yen C.T., Wu W.J., Chen Y.T., Chang W.C., Yang S.H., Shen S.Y., Su J., Chen H.Y. (2021). Surgical resection of brain metastases prolongs overall survival in non-small-cell lung cancer. Am. J. Cancer Res..

[9] Patil C.G., Pricola K., Sarmiento J.M., Garg S.K., Bryant A., Black K.L. (2017). Whole brain radiation therapy (WBRT) alone versus WBRT and radiosurgery for the treatment of brain metastases. Cochrane Database Syst. Rev..

[10] Zhao B., Wang Y., Wang Y., Chen W., Zhou L., Liu P.H., Kong Z., Dai C., Wang Y., Ma W. (2020). Efficacy and safety of therapies for EGFR-mutant non-small cell lung cancer with brain metastasis: An evidence-based Bayesian network pooled study of multivariable survival analyses. Aging.

[11] Thomas R., Srivastava S., Katreddy R.R., Sobieski J., Zhang W. (2019). Kinase-inactivated EGFR is required for the survival of wild-type EGFR-expressing cancer cells treated with tyrosine kinase inhibitors. Int. J. Mol. Sci..

[12] Rosell R., Carcereny E., Gervais R., Vergnenegre A., Massuti B., Felip E., Palmero R., Garcia-Gomez R., Pallares C., Sanchez J.M. (2012). Erlotinib versus standard chemotherapy as first-line treatment for European patients with advanced EGFR mutation-positive non-small-cell lung cancer (EURTAC): A multicentre, open-label, randomised phase 3 trial. Lancet Oncol..

[13] Mitsudomi T., Morita S., Yatabe Y., Negoro S., Okamoto I., Tsurutani J., Seto T., Satouchi M., Tada H., Hirashima T. (2010). Gefitinib versus cisplatin plus docetaxel in patients with non-small-cell lung cancer harbouring mutations of the epidermal growth factor receptor (WJTOG3405): An open label, randomised phase 3 trial. Lancet Oncol..

[14] Yun P.J., Wang G.C., Chen Y.Y., Wu T.H., Huang H.K., Lee S.C., Chang H., Huang T. (2019). Brain metastases in resected non-small cell lung cancer: The impact of different tyrosine kinase inhibitors. PLoS ONE.

[15] Zeng Y.D., Liao H., Qin T., Zhang L., Wei W.D., Liang J.Z., Xu F., Dinglin X., Ma S., Chen L. (2015). Blood-brain barrier permeability of gefitinib in patients with brain metastases from non-small-cell lung cancer before and during whole brain radiation therapy. Oncotarget.

[16] Clarke J.L., Pao W., Wu N., Miller V.A., Lassman A.B. (2010). High dose weekly erlotinib achieves therapeutic concentrations in CSF and is effective in leptomeningeal metastases from epidermal growth factor receptor mutant lung cancer. J. Neurooncol..

[17] Leonetti A., Sharma S., Minari R., Perego P., Giovannetti E., Marcello T. (2019). Resistance mechanisms to osimertinib in EGFR-altered non-small cell lung cancer. Br. J. Cancer.

[18] Ballard P., Yates J.W., Yang Z., Kim D.W., Yang J.C., Cantarini M., Pickup K., Jordan A., Hickey M., Grist M. (2016). Preclinical Comparison of Osimertinib with Other EGFR-TKIs in EGFR-Mutant NSCLC Brain Metastases Models, and Early Evidence of Clinical Brain Metastases Activity. Clin. Cancer Res..

[19] Ramalingam S.S., Vansteenkiste J., Planchard D., Cho B.C., Gray J.E., Ohe Y., Zhou C., Reungwetwattana T., Cheng Y., Chewaskulyong B. (2020). Overall Survival with Osimertinib in Untreated. N. Engl. J. Med..

[20] Soria J.C., Ohe Y., Vansteenkiste J., Reungwetwattana T., Chewaskulyong B., Lee K.H., Dechaphunkul A., Imamura F., Nogami N., Kurata T. (2018). Osimertinib in Untreated EGFR-altered Advanced Non-Small-Cell Lung Cancer. N. Engl. J. Med..

[21] Goss G., Tsai C.M., Shepherd F.A., Ahn M.J., Bazhenova L., Crinò L., de Marinis F., Felip E., Morabito A., Hodge R. (2018). CNS response to osimertinib in patients with T790M-positive advanced NSCLC: Pooled data from two phase II trials. Ann. Oncol..

[22] Wu Y.L., Ahn M.J., Garassino M.C., Han J.Y., Katakami N., Kim H.R., Hodge R., Kaur P., Brown A.P., Ghiorghiu D. (2018). CNS Efficacy of Osimertinib in Patients With T790M-Positive Advanced Non-Small-Cell Lung Cancer: Data From a Randomized Phase III Trial (AURA3). J. Clin. Oncol..

[23] Yamaguchi H., Wakuda K., Fukuda M., Kenmotsu H., Mukae H., Ito K., Chibana K., Inoue K., Miura S., Tanaka K. (2021). A Phase II Study of Osimertinib for Radiotherapy-Naive Central Nervous System Metastasis From NSCLC: Results for the T790M Cohort of the OCEAN Study (LOGIK1603/WJOG9116L). J. Thorac. Oncol..

[24] Yang J.C.H., Kim S.W., Kim D.W., Lee J.S., Cho B.C., Ahn J.S., Lee D.H., Kim T.M., Goldman J.W., Natale R.B. (2020). Osimertinib in Patients With Epidermal Growth Factor Receptor Mutation-Positive Non-Small-Cell Lung Cancer and Leptomeningeal Metastases: The BLOOM Study. J. Clin. Oncol..

[25] Singhi E.K., Horn L., Sequist L.V., Heymach J., Langer C.J. (2019). Advanced Non-small Cell Lung Cancer: Sequencing Agents in the EGFR-altered/ALK-Rearranged Populations. Am. Soc. Clin. Oncol. Educ. Book.

[26] Huang Y.H., Hsu K.H., Tseng J.S., Yang T.Y., Chen K.C., Su K.Y., Yu S.L., Chen J.W., Chang J.C. (2022). The Difference in Clinical Outcomes Between Osimertinib and Afatinib for First-Line Treatment in Patients with Advanced and Recurrent EGFR-Mutant Non-Small Cell Lung Cancer in Taiwan. Target. Oncol..

[27] Gen S., Tanaka I., Morise M., Koyama J., Kodama Y., Matsui A., Hase T., Hibino Y., Yokoyama T., Kimura T. (2022). Clinical efficacy of osimertinib in EGFR-mutant non-small cell lung cancer with distant metastasis. BMC Cancer.

[28] Zhao Y., Li S., Yang X., Chu L., Wang S., Tong T., Chu X., Yu F., Zeng Y., Guo T. (2022). Overall survival benefit of osimertinib and clinical value of upfront cranial local therapy in untreated EGFR-mutant non-small cell lung cancer with brain metastasis. Int. J. Cancer.

[29] Zhou Y., Wang B., Qu J., Yu F., Zhao Y., Li S., Zeng Y., Yang X., Chu L., Chu X. (2020). Survival outcomes and symptomatic central nervous system (CNS) metastasis in EGFR-mutant advanced non-small cell lung cancer without baseline CNS metastasis: Osimertinib vs. first-generation EGFR tyrosine kinase inhibitors. Lung Cancer.

[30] Reungwetwattana T., Nakagawa K., Cho B.C., Cobo M., Cho E.K., Bertolini A., Bohnet S., Zhou C., Lee K., Nogami N. (2018). CNS Response to Osimertinib Versus Standard Epidermal Growth Factor Receptor Tyrosine Kinase Inhibitors in Patients With Untreated EGFR-altered Advanced Non-Small-Cell Lung Cancer. J. Clin. Oncol..

[31] Liang H., Liu X., Wang M. (2018). Immunotherapy combined with epidermal growth factor receptor tyrosine kinase inhibitors in non-small-cell lung cancer treatment. Oncol. Targets Ther..

